# Should We Acknowledge ChatGPT as an Author?

**DOI:** 10.2188/jea.JE20230078

**Published:** 2023-07-05

**Authors:** Atsushi Goto, Kota Katanoda

**Affiliations:** 1 Department of Public Health, School of Medicine, Yokohama City University, Yokohama, Japan; 2 Department of Health Data Science, Graduate School of Data Science, Yokohama City University, Yokohama, Japan; 3 Division of Surveillance and Policy Evaluation, National Cancer Center Institute for Cancer Control, Chuo-ku, Tokyo, Japan

In 2020, large language models using artificial intelligence (AI) represented by GPT-3 (Generative Pretrained Transformer - 3) appeared,^1^ making a solid step towards Artificial General Intelligence (AGI), an intelligence that can learn or comprehend any intellectual job that a human can accomplish. Open AI, an American AI research laboratory, made a breakthrough innovation using the technology of GPT-3 and launched ChatGPT in November 2022. ChatGPT is an artificial intelligence (AI) chatbot that generates coherent sentences by analyzing the statistical patterns found in a large database of text extracted from the Web.^2^ This has had a major impact on the publishing industry, education, and science. There is no doubt that virtually every area of human writing will eventually involve AI technology, either explicitly or implicitly.

As an online print paper in the *Journal of Epidemiology*, a letter to the editor on this topic has been published as a timely and well-thought-out insight about ChatGPT’s authorship.^3^ The letter concluded, in accordance with the authorship guidelines recommended by the IMCJE (the International Committee of Medical Journal Editors),^4^ that ChatGPT is not qualified as an author because it cannot approve the final manuscript nor take responsibility for manuscript content. Having received this letter, we conducted a simple survey among our Editorial Board members, asking them about the potential role of ChatGPT and the authors’ responsibilities. None of the respondents thought that it could be an author. The majority (74%) thought that ChatGPT could be used as a tool, among whom 63% thought that it should be disclosed during submission. Although officially unstated in our Guide for Authors,^5^ we, as editors of the *Journal of Epidemiology*, agree that ChatGPT should not be acknowledged as an author of scientific papers. As mentioned in the letter cited above,^3^ the Science Family of Journals banned the use of AI technologies without explicit permission from the editors,^6^ while some other publishing groups subsequently announced a policy allowing the use of AI as a tool (not as an author) under the condition of appropriate disclosure.^7^^,^^8^

Additional issues need to be considered beyond deciding whether to treat ChatGPT as an author. Of greatest concern, ChatGPT can cause fatal errors. Scientific papers should make inferences based on facts. However, the words written by ChatGPT are not necessarily facts; it often writes incorrect sentences. We provide several examples where ChatGPT has offered an incorrect response in the field of epidemiology. For example, when we asked, “Please tell me relevant citations to evaluate the association of coffee intake with liver cancer risk in Japan.”, it offered the following as a relevant citation on February 20, 2023: “Inoue et al. Coffee and green tea consumption and the risk of liver cancer in Japan: the Japan Public Health Center-based Prospective Study. Cancer Causes Control. 2009;20(5): 5 – 15. doi:10.1007/s10552-008-9235-5…”.^2^ Unfortunately, there is no paper with the title, and the doi indicates an unrelated paper written by different authors. Furthermore, when we asked, “What are the top causes of death in Japan in 2020?”, it answered as follows on March 20, 2023: “According to the Ministry of Health, Labour and Welfare in Japan, the top three causes of death in 2019 were: 1. Cancer: 29.5% of deaths, 2. Heart disease: 15.1% of deaths, 3. Pneumonia: 8.4% of deaths”.^2^ However, the actual top causes of death in 2019 were malignant neoplasms (27.3%), heart diseases (15.0%), and senility (8.8%).^9^ ChatGPT incorrectly reported pneumonia as the third cause of death. A quick Internet search can detect that these sentence are false, but ChatGPT does not seem to judge them. Many of today’s ChatGPT sources are available on the Internet but have not undergone rigorous critical scrutiny. Thus, there is a risk that uncertain information or data will be treated as facts. Therefore, it is the responsibility of the authors to confirm that the texts written by ChatGPT are correct, and the scientific community is responsible for monitoring this. Another AI chatbot, Perplexity AI,^10^ has recently drawn considerable attention because it sometimes provides more accurate answers and information sources than ChatGPT. However, it is unlikely that ChatGPT or Perplexity AI will satisfy the ICMJE requirements of authorship.

Technologies such as ChatGPT are expected to advance significantly in the future. Indeed, Open AI has recently released GPT-4, a newer version of ChatGPT, on March 14, 2023, which seems to be more reliable and able to handle more complex instructions than the earlier version.^11^ We hope that by recognizing the chatbot’s shortcomings and using the chatbot effectively, scientific evidence will be efficiently published, and science will progress.
